# Caffeine ingestion compromises thermoregulation and does not improve cycling time to exhaustion in the heat amongst males

**DOI:** 10.1007/s00421-024-05460-z

**Published:** 2024-04-03

**Authors:** Kevin John, Sayyam Kathuria, Jenny Peel, Joe Page, Robyn Aitkenhead, Aimee Felstead, Shane M. Heffernan, Owen Jeffries, Jamie Tallent, Mark Waldron

**Affiliations:** 1 Research Institute for Sport and Exercise, University of Canberra, Canberra, Australia; 2 Applied Sports Science Technology and Medicine Research Centre (A-STEM), Faculty of Science and Engineering, Bay Campus, Swansea University, Swansea, Wales SA1 8EN UK; 3 School of Biomedical, Nutritional and Sport Sciences, Newcastle University, Newcastle Upon Tyne, UK; 4 School of Sport, Rehabilitation, and Exercise Sciences, University of Essex, Colchester, UK; 5 Department of Physiotherapy, Faculty of Medicine, Nursing and Health Sciences, School of Primary and Allied Health Care, Monash University, Clayton, Australia; 6 Welsh Institute of Performance Science, Swansea University, Swansea, UK; 7 School of Health and Behavioural Sciences, University of the Sunshine Coast, Sippy Down, QLD Australia

**Keywords:** Caffeine supplementation, Ergogenic, Endurance performance, Heat, Thermoregulation

## Abstract

**Purpose:**

Caffeine is a commonly used ergogenic aid for endurance events; however, its efficacy and safety have been questioned in hot environmental conditions. The aim of this study was to investigate the effects of acute caffeine supplementation on cycling time to exhaustion and thermoregulation in the heat.

**Methods:**

In a double-blind, randomised, cross-over trial, 12 healthy caffeine-habituated and unacclimatised males cycled to exhaustion in the heat (35 °C, 40% RH) at an intensity associated with the thermoneutral gas exchange threshold, on two separate occasions, 60 min after ingesting caffeine (5 mg/kg) or placebo (5 mg/kg).

**Results:**

There was no effect of caffeine supplementation on cycling time to exhaustion (TTE) (caffeine; 28.5 ± 8.3 min *vs*. placebo; 29.9 ± 8.8 min, *P* = 0.251). Caffeine increased pulmonary oxygen uptake by 7.4% (*P* = 0.003), heat production by 7.9% (*P* = 0.004), whole-body sweat rate (WBSR) by 21% (*P* = 0.008), evaporative heat transfer by 16.5% (*P* = 0.006) and decreased estimated skin blood flow by 14.1% (*P* < 0.001) compared to placebo. Core temperature was higher by 0.6% (*P* = 0.013) but thermal comfort decreased by − 18.3% (*P* = 0.040), in the caffeine condition, with no changes in rate of perceived exertion (*P* > 0.05).

**Conclusion:**

The greater heat production and storage, as indicated by a sustained increase in core temperature, corroborate previous research showing a thermogenic effect of caffeine ingestion. When exercising at the pre-determined gas exchange threshold in the heat, 5 mg/kg of caffeine did not provide a performance benefit and increased the thermal strain of participants.

## Introduction

Caffeine (1,3,7-trimethylxanthine) is a widely used stimulant, consumed by 80% of the world’s population, most commonly via plant sources (coffee, tea etc.) or manufactured products, such as energy drinks, caffeinated gums and gels (Burke 2008; Ogawa and Ueki 2007). Owing to the molecular similarity between caffeine and adenosine, caffeine acts as an adenosine receptor antagonist in central and peripheral locations. This opposes the established effects of adenosine, such as decreased arousal, inhibition of excitatory neurotransmitters and increased sleep or drowsiness (Fredholm et al. 1999). Further, the competitive blockage of adenosine binding by caffeine has been reported to reduce pain perception and increase dopaminergic transmission, which have been linked to physical-performance enhancement (Tarnopolsky 2008). Indeed, as a result of the rapid post-ingestion bioavailability (1-h; Liguori et al. 1997) and its consistent ergogenic effect amongst athletic populations (Sökmen et al. 2008; Spriet 1995), caffeine has been identified as a ‘primary’ ergogenic aid, strongly supported by the International Olympic Committee (IOC; Maughan et al. 2018). Consistent with the above evidence, 76% of elite athletes consume caffeine prior to or during exercise, particularly those competing in endurance events (Aguilar-Navarro et al. 2019).

Whilst there is an overwhelming body of evidence to support caffeine’s ergogenicity in regard to endurance performance (Ganio et al. 2009; Southward et al. 2018), the majority of studies have been conducted in thermoneutral environmental conditions (Guest et al. 2021). Given that exercise in the heat exacerbates cardiovascular strain (González-Alonso et al. 1999), causes impairment in volunrary activation (Nybo and Nielsen 2001) and increases demand on fluid-regulatory processes (Sawka and Montain 2000) compared to thermoneutral conditions, it is reasonable to question the administration of a potent stimulant prior to entering a thermally stressful environment. This is despite caffeine supplementation being common place amongst athletes competing in the heat (Desbrow and Leveritt 2006) and other thermo-physiologically demanding occupations e.g. mining and firefighting (Berkowsky et al. 2022; Peetz et al. 2012). Indeed, caffeine supplementation studies in the heat have been inconclusive, with some showing improvements ranging between 3 and 30% (Beaumont and James 2017; Ganio et al. 2011; Ping et al. 2010) and others reporting no effect on endurance performance (Cheuvront et al. 2009; Cohen 1996; Hanson et al. 2019; Roelands et al. 2011; Suvi et al. 2016). However, evidence drawn from meta-analyses has demonstrated caffeine has no ergogenic effect on endurance exercise in the heat, but can significantly increase core temperature (*T*_core_) compared to placebo trials (Peel et al. 2021). The null effects of caffeine on performance were supported more recently via a further meta-analysis (Naulleau et al. 2022), where it was also reported that exercising *T*_core_ appears to increase at significantly quicker rates following caffeine consumption (~ 0.10 °C/h) compared to placebo. Collectively, these meta-analytical findings indicate a progressive reduction in heat storage capacity following caffeine supplementation during or prior to exercise in the heat, which has many concerning health and performance implications.

Evidence provided across a number of studies indicates the potential physiological reasons for increased *T*_core_ following caffeine ingestion in the heat. For example, a high dose of caffeine (9 mg/kg) resulted in a significantly higher (8 W) metabolic heat production ($\dot{H}$_prod_) during exercise in the heat (40 °C, 25% relative humidity [RH]) compared to placebo (Ely et al. 2011). The above trend in $\dot{H}$_prod_ was paralleled by greater mean body temperatures, despite controlling for external work at a pre-determined fraction of aerobic capacity (30 min at 50% $\dot{V}$ O_2peak_). Using a different method to control exercise intensity, Hunt et al. (2021) recently reported *a* ~ 0.3 °C increase in esophageal temperature, alongside substantial (~ 30%) reductions in skin blood flow (arm and back) whilst exercising at fixed $\dot{H}$_prod_ (7 W/kg) for 60 min in dry heat (30 °C and 30% RH), following caffeine (5 mg/kg) consumption. These results are consistent with the reported widespread mechanisms of caffeine, such as increased basal metabolic rate (Dulloo et al. 1989) and exercising heart rate (Stebbins et al. 2001), alongside reductions in cutaneous blood flow (Daniels et al. 1998). This could collectively explain the greater $\dot{H}$_prod_ and rise in *T*_core_ in the absence of performance improvements in the heat. Although, supplementing with lower doses of caffeine (3 mg/kg) have resulted in conflicting results for *T*_core_ responses during exercise in the heat (Ganio et al. 2011; Roelands et al. 2011; Beaumont and James 2017). However, it is important to consider that the majority of studies investigating thermoregulatory effects of caffeine consumption during prolonged exercise in the heat have controlled intensity using fractions of pre-determined power output at the thermoneutral $\dot{V}$ O_2peak/max_ (Ely et al. 2011; Ganio et al. 2011; Juan et al. 2009; Roelands et al. 2011; Stebbins et al. 2001). Whilst this is practically convenient, the reported heterogeneity in physiological responses between individuals exercising at the same relative intensity when determined in this manner (Iannetta et al. 2020; Lansley et al. 2011; Scharhag-Rosenberger et al. 2010), means that adopting this approach to establish intensities in the heat is likely to provide less certainty regarding the physiological domain of exercise. This might be exacerbated when transferring pre-determined thermoneutral measures to a hot environment, where the change in relative intensity could compound the effect. This may lead to inconsistent metabolic demand, and thus unknown levels of $\dot{H}$_prod_, which is a known driver of thermoregulatory effectors, such as sweat gland recruitment and sweat production (Cramer and Jay 2015; Jay et al. 2011). It is possible that prescription of exercise intensity using exercise domains might help to control the exercise intensity within- and between-individuals (Iannetta et al. 2020; Meyler et al. 2023) and remove the risk of heterogenous physiological responses, which could be masking a thermogenic effect of caffeine whilst exercising in the heat.

Accordingly, the current study aimed to investigate the effects of an acute dose of caffeine (5 mg/kg) on: (i) TTE at the pre-determined thermoneutral gas exchange threshold (GET) and (ii) thermo-physiological responses (i.e. body temperature, whole-body calorimetric components, metabolic, cardiovascular and perceptual) to exercise in a hot environment (~ 35 °C and  ~ 40% RH). We hypothesised that acute caffeine ingestion prior to exercise in the heat would reduce TTE and increase the thermo-physiological response to exercise across the measured variables.

## Methods

### Participants

Twelve healthy males (age 23 ± 4 years; body mass 80 ± 6 kg; stature 1.85 ± 0.05 m; $\dot{V}$ O_2peak_ 48.9 ± 4.7 mL/kg/min) volunteered to participate in the study. Inclusion criteria for this study were: healthy (absence of acute or chronic illness, as determined at pre-screening), recreationally active (exercising aerobically for a minimum of three times per week for more than 30 min), males or females, aged between 18 and 40 years, no heat acclimation or acclimatisation activities (such training in the heat or visiting a hot country in the past three months). The participants were mostly amateur team sports players (10/12), with some (2/10) taking part in continuous endurance exercise only. We identify them as tier 1 athletes (Mckay et al. 2022). A-priori sample size was calculated using G*Power (Version 3.1.9.6), based on previously reported changes in TTE performance following acute caffeine supplementation in the heat (Cohen’s *d* = 1.02, Ping et al. 2010). In a within-participants design, a sample of 10 participants was deemed sufficient to identify differences between conditions, with a power of 0.80 and $\alpha$ = 0.05. To account for potential dropouts the study over-recruited by two participants. The participants completed a manual, written food diary 1 day before the first experimental trial and were asked to replicate it prior to the second experimental trial. Participants were instructed to drink 500 mL of fluid in the 2 h before exercise and a further 200 mL 20 min before coming to the laboratory. The foods consumed were not weighed but estimated approximately by the participants, who were encouraged to take images of their food to facilitate replication on the cross-over trial. All participants reported successfully copying their diet on the second visit. A member of the research team maintained contact with participants to encourage adherence to the above instructions. Participants were also required to refrain from alcohol and caffeine for 24 h and avoid strenuous exercise 48 h prior to testing. Participants were also provided with a list of caffeine sources to refrain from consuming across the 24 h prior to each visit. All participants were requested to stop taking any dietary supplementation and avoid the use of saunas or hot water immersion for at least 1-week before and the entire duration of the study. Institutional ethical approval was provided for this study, which was conducted in accordance with the World Medical Association (2013), apart from pre-registration on a public database.

### Design

The study adopted a double-blinded, counter-balanced, placebo-controlled cross-over design. After initial health-screening, all participants reported to the laboratory on three separate occasions, each separated by a maximum of 7 days and a minimum of 2 days to minimise any acclimation effects. The first visit consisted of preliminary testing and familiarisation. On visits 2 and 3, participants completed the experimental trials, ingesting caffeine or placebo 1 h before commencing exercise. The order in which participants completed the caffeine and placebo trials was randomised using an online software, with the 50% of participants in one blinded condition and the remainder taking part in the second blinded condition, before crossing over (Urbaniak and Plous 2015). To prevent investigator bias, the randomisation was completed by an independent person, who was not involved in data collection. The independent person was also responsible for encapsulating the caffeine and placebo supplements and randomly allocating them to participants. During pre-screening, a questionnaire was provided to participants to determine their caffeine intake, with ≥ 100 mg/day of caffeine day considered ‘habitual’ consumer (Hunt et al. 2021). In the current study, all participants qualified as habitual consumers.

### Preliminary testing

During visit 1, all participants completed an incremental ramp test to volitional exhaustion on a mechanically braked cycle ergometer (Monark Exercise AB, Ergomedic 874E, Varberg, Sweden) in thermoneutral conditions (20.0 ± 2.1 °C) to determine peak oxygen consumption ($\dot{V}$ O_2peak_) and the power output at GET. Participants first completed a 5 min warm-up, cycling at 80 W, followed by a 5 min resting period. The incremental test started at a workload of 120 W and increased by approximately 24 W/min at a fixed cadence of 80 rev/min until volitional exhaustion or when the cadence dropped below 70 rev/min for more than 20 s. Pulmonary gas was recorded using a breath-by-breath gas analyser (Vyntus CPX, Carefusion Germany 234 GmbH). Prior to every testing visit, the gas analyser system was calibrated using known calibration gases (15.94% O_2_, 5.00% CO_2,_ BAL. N_2_), as well as measured ambient gas fractions. The turbine transducer was volume-calibrated automatically by the system, using flow rates 2 L/s and 0.2 L/s. Heart rate (HR) was recorded throughout the trial (Polar Heart Rate Monitor M400, Warwick, UK). The $\dot{V}$ O_2peak_ was calculated by measuring the highest 30 s average pulmonary oxygen uptake ($\dot{V}$ O_2_). Breath-by-breath pulmonary carbon dioxide production ($\dot{V}$ CO_2_) and $\dot{V}$ O_2_ data from the incremental ramp test was plotted to determine GET, using both the ventilatory equivalents (Beaver et al. 1986) and simplified v-slope method (Schneider et al. 1993), with power at the GET adjusted for 2/3 ramp rate. After a 15 min rest period, participants completed a TTE familiarisation trial, which required them to cycle at the power output associated with thermoneutral GET in an environmental chamber mimicking experimental conditions (~ 35 °C,  ~ 40% RH), without fanning or convective air flow. The above method of exercise intensity prescription was selected, as it demarcates a boundary between moderate and heavy domains (Poole and Jones 2012), which has been reported to be more appropriate for monitoring physiological responses to exercise (Poole et al. 2021), and has been used for sub-maximal assessment of exercise tolerance, whilst sufficiently increasing $\dot{H}$_prod_ and other thermoregulatory responses (Page et al. 2019; Fowler et al. 2021). Across all participants, the GET occurred at 61 ± 5% of the thermoneutral $\dot{V}$ O_2peak_. During the subsequent TTEs in a hot environment, the participants cycled at 70 ± 3 and 74 ± 3% of the thermoneutral $\dot{V}$ O_2peak_ for the placebo and caffeine conditions, respectively.

### Experimental trials

All subsequent tests were conducted in the heat (~ 35 °C,  ~ 40% RH). Participants arrived at the laboratory 1 h prior to testing and were instructed to insert a rectal probe 10 cm past the anal sphincter to measure baseline *T*_core_. A urine sample was also taken to determine hydration status using a refractometer (Osmochek, Vitech Scientific Ltd, West Sussex, UK). A reading of > 600 mOsm/kg/H_2_O indicated the threshold for hypohydration, in which the participant consumed 500 mL of water and was required to wait 30 min prior to testing. The participants pre-body mass was measured (MPMS-230, Marsden Weighing Group, Oxfordshire, UK) wearing cycling shorts with the rectal probe inserted, prior to entering the environmental chamber. The scales were accurate to 50 g and the same cycling shorts were worn during measurements to permit some privacy for participants in the laboratory. The shorts and rectal probes were weighed before and after measurements and accounted for in the final body mass measurement.

Participants then entered the environmental chamber and rested in a seated position on the cycle ergometer for 5 min. During this period, skin thermistors (Grant Instruments Ltd., Cambridge, UK) were attached to four sites on the participant’s right side: upper chest, mid-humerus, mid-thigh and mid-calf. Core and skin temperature were recorded continuously via a Squirrel data logger (SQ2010; Grant Instruments Ltd., Cambridge, UK). Mean skin temperature (*T*_skin_) was calculated using Ramanathan’s weighted equation (Ramanathan 1964):

1 $$T_{skin} = \, 0.3 \times (T_{chest} + T_{arm} ) + 0.2 \times (T_{thigh} + T_{calf} )$$

Participants were fitted with a face mask and monitored for their pulmonary breath-by-breath responses using the same gas analyser throughout the TTE (Vyntus CPX, Carefusion Germany 234 GmbH). HR was monitored continuously and combined with $\dot{V}$ O_2_ data to derive the oxygen pulse [O_2_ pulse; mL pulmonary $\dot{V}$ O_2_ (mL/min)/HR (beats/min)]. Participants were then instructed to begin the experimental trial and were required to maintain a cadence of 80 rev/min at an intensity equivalent to thermoneutral GET until volitional exhaustion. Total carbohydrate and fat oxidation rates (g/min) were calculated using equations suggested for moderate-to-high intensity exercise but less than approximately 75% of $\dot{V}$ O_2max_ (Jeukendrup and Wallis 2005), with the assumption of negligible protein oxidation. The participants were informed to abstain from consuming fluid until the end of the test. The participants were supervised constantly during the trials by a member of the research team and verbal feedback was provided to maintain the intended cadence within tolerance of the participant’s control. Exhaustion was defined as the time point at which the participant voluntarily withdrew or when pedal cadence dropped below 70 rev/min for more than 20 s. The coefficient of variation for this test in our laboratory is 3.8% whilst cycling in the heat. This form of test was chosen based on its reliability amongst recreationally trained participants, without specific cycling training, for relatively short periods of time in the heat. Rating of perceived exertion (RPE) was recorded on a 6–20-point Borg scale (Borg 1982). Thermal comfort (TC) was recorded on a 7-point scale where − 3 = “much too cool”, 0 = “comfortable” and 3 = “much too warm” (Bedford 1936). RPE and TC were recorded at rest, and every 3 min during the experimental trial and at completion. Post-body mass was recorded in the same manner as pre-measures, with the change per unit time (min) from pre-body mass measures used as an indication of WBSR. The participants were then advised to consume adequate fluids and foods to aid in recovery.

### Partitional calorimetry

Participant’s *Ḣ*_prod_ during the TTE was determined by subtracting the rate of mechanical work from the cycle ergometer ($\dot{{\text{W}}}$ k) from the rate of metabolic energy expenditure (Ṁ; Eq. 1):

2 $$\dot{H}_{prod} = \dot{M} - Wk [W]$$

where metabolic energy expenditure (Ṁ) was determined using measured $\dot{{\text{V}}}$ O_2_ and respiratory-exchange ratio (RER) in the 10% epoch of each TTE (Eq. 3):s

3 $$\dot{M} = \dot{V}O_{2} \times \frac{{\left( {\left( {\frac{RER - 0.7}{{0.3}}} \right) \times 21.13} \right) + \left( {\left( {\frac{1.0 - RER}{{0.3}}} \right) \times 19.62} \right)}}{60} \times 1000 \left[ W \right]$$

The *Ḣ*_prod_ (W/m^2^) was expressed relative to the participants’ body surface area (BSA; Eqs. 4 and 5; [Cramer and Jay 2014]).

4 $$\dot{H}_{{{\text{prod}}}} = \frac{{\dot{H}_{{{\text{prod}}}} }}{{{\text{BSA}}}} [{\text{W}}/{\text{m}}^{2} ]$$

5 $${\text{BSA}} = 0.00718 \times ({\text{body mass }}\left( {{\text{kg}}} \right)^{0.425} ) \times \left( {{\text{height }}({\text{cm}})^{0.725} } \right) [{\text{m}}^{2} ]$$

Dubois and Dubois equation [Du Bois and Du Bois 1916])

On the assumption that blood entering and leaving the cutaneous circulation was equal to core and skin temperatures, respectively, estimated skin blood flow (SkBF) in the first and final 50% of the TTE trial was determined as (Sawka and Young 2006).

6 $${\text{SkBF}} = \frac{{\left( {\frac{1}{{{\text{SH}}}} \cdot {\text{H}}_{{{\text{prod}}}} } \right)}}{{\left( {T_{core } - T_{skin} } \right)}}$$

where SH = specific heat of the blood (~ 1 kcal °C^−1^) and *Ḣ*_prod_ is expressed in kcal min^−1^. *T*_core_ and *T*_skin_ where taken as the mean measurements during the first and final 50% of the TTE to provide an estimated SkBF measure.

Evaporative heat transfer at the skin surface ($\dot{E}$_sk_) was determined as:

7 $$\dot{E}sk = \frac{\Delta BM \times \lambda / t }{{{\text{BSA}}}} [W/m^{2} ]$$

where change in body mass ($\Delta {\text{BM}};\mathrm{ g}$) is multiplied by the latent heat of vaporisation ($\lambda$; 2426 J/g) and divided by time (*t*; s) and BSA.

### Caffeine supplementation and plasma caffeine analysis

All supplements were acquired in white anhydrous powder form and were separated into gelatine capsules using analytical balance scales (Ohaus, Navigator N24120, Nänikon Switzerland). The participants body mass recorded during visit 1 was used to measure correct doses, such that supplements were balanced and equal number of capsules were ingested by the participants between trials 2 and 3. The capsules contained either caffeine (5 mg/kg BM; Blackburn Distributions Ltd., Burnley, UK) or a placebo (Maltodextrin, My Protein, Manchester, UK) and were administered 1 h prior to exercise after preparation using an analytical balance with a measurement resolution of 0.01 g (Ohaus, Navigator N24120, Nänikon Switzerland. This timeframe was chosen to ensure peak-plasma appearance of caffeine following oral capsule ingestion (Liguori et al. 1997; Newton et al. 1981). In 10 of the participants, blood draws (5 mL) were taken 50 min after caffeine ingestion, which were subsequently stored at −  80 °C and later thawed and centrifuged at 3000 *g* for 10 min for analysis of plasma caffeine concentration using a high-performance liquid chromatography technique.

### Statistical analysis

The normality of the residuals was assessed using the Shapiro–Wilk test, after which two-way repeated measures analysis of variance was conducted, with the effects of condition (caffeine or placebo) and time (10% epochs across the TTE or relevant time points) on outcome variables ($\dot{V}$ O_2_, Glucose and fat oxidation rates, RPE, HR, O_2_ pulse, $\dot{H}$_prod_, *T*_core_ and *T*_skin_). It was deemed suitable to analyse data in 10% epochs, given the minor differences in TTE duration across participant, thus facilitating like-for-like comparisons. Utilising 10% epochs across shorter duration TTEs (~ 30 min) also reduces the chance of overlooking smaller absolute differences in variables across time. This was verified during the analysis process. A Greenhouse–Geisser correction was applied when the assumption of sphericity was violated. Where interaction effects were found, *post-hoc* analysis was performed with Bonferroni corrections to identify pairwise differences. Analysis of residuals from the TC data demonstrated consistent non-normal distribution and was, therefore, analysed using the non-parametric aligned ranks test for repeated measures (‘*ARTool’* package in RStudio), with the *apriori* intention to perform *post-hoc* tests on interaction effects. For the purpose of statistical analysis, the TC and RPE measures were aggregated across three epochs (beginning, middle and end) across the trial to account for the discontinuous measurements. Two-tailed paired samples *t* tests was used to identify significant differences between the performance trials, as well as the differences in the WBSR, baseline *T*_core_ and the rate of rise in *T*_core_ between baseline and the start of the TTE. Similarly, paired samples *t* tests were also used to assess trial order effects of the primary outcome performance measure (TTE), as well as the mean *T*_core,_ WBSR and $\dot{H}$_prod_ across the exercise trials, which were selected as markers of the thermoregulatory response to exercise. Statistical significance was set at *P* < 0.05 and all analyses, besides the non-parametric tests, were performed in IBM SPSS Statistics (Version 21; IBM Corp., Armonk, NY, USA). Cohen’s *d* for repeated measures was calculated as the mean difference divided by the standard deviation of the differences to interpret the effect of pairwise changes.

## Results

### Time-to-exhaustion and plasma caffeine analysis

**Fig. 1:**
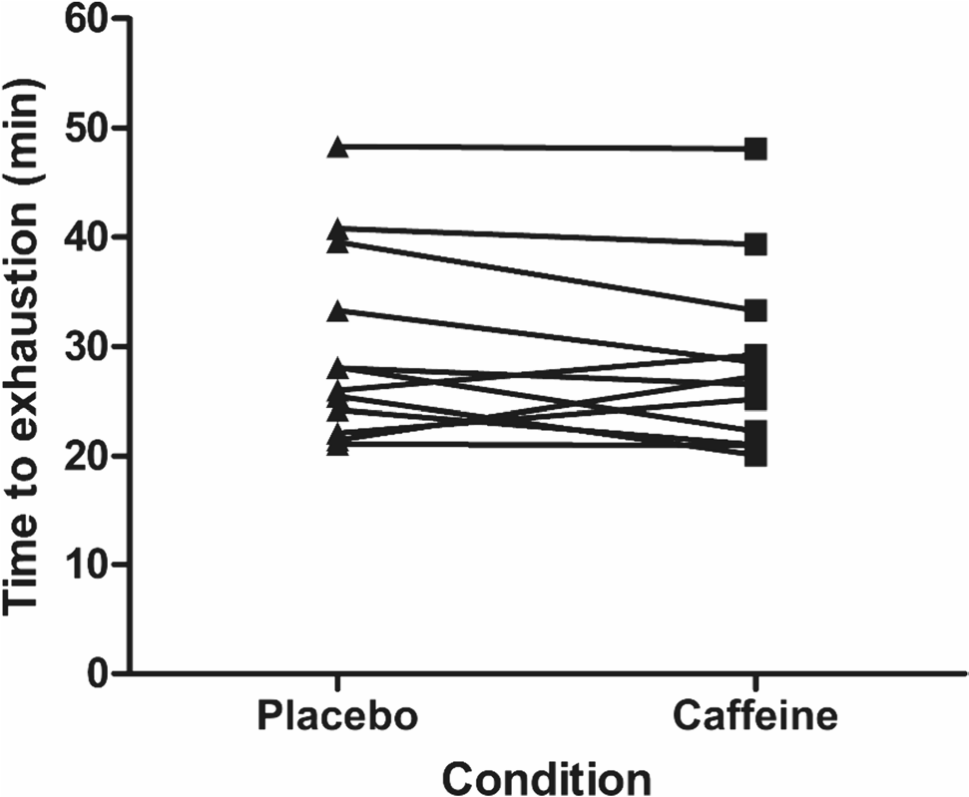
Time-to-exhaustion at the gas exchange threshold during exercise in the heat in placebo and caffeine conditions

There was no effect of caffeine supplementation on TTE (*t*_(11)_ = − 1.2, *P* = 0.251, *d* = 0.16; Fig. 1). The placebo condition cycled for 29.9 ± 8.8 min, whereas the caffeine condition cycled for 28.5 ± 8.3 min. There was a higher plasma caffeine concentration in the caffeine condition compared to the placebo (caffeine 33.1 ± 4.5 µM *vs.* placebo 1.4 ± 1.9 µM; *d* = 9.2); however, caffeine was undetectable in seven of the ten placebo samples, so no further statistics are reported. There were no trial order effects for TTE (*t*_(11)_ = 1.12, *P* = 0.286, *d* = 0.15), $\dot{H}$_prod_ (*t*_(11)_ = − 0.2, *P* = 0.827, *d* = 0.08), *T*_core_ (*t*_(11)_ = 0.29, *P* = 0.775, *d* = 0.08) and WBSR (*t*_(11)_ = 0.44, *P* = 0.669, *d* = 0.16).

### Cardiometabolic responses

**Fig. 2:**
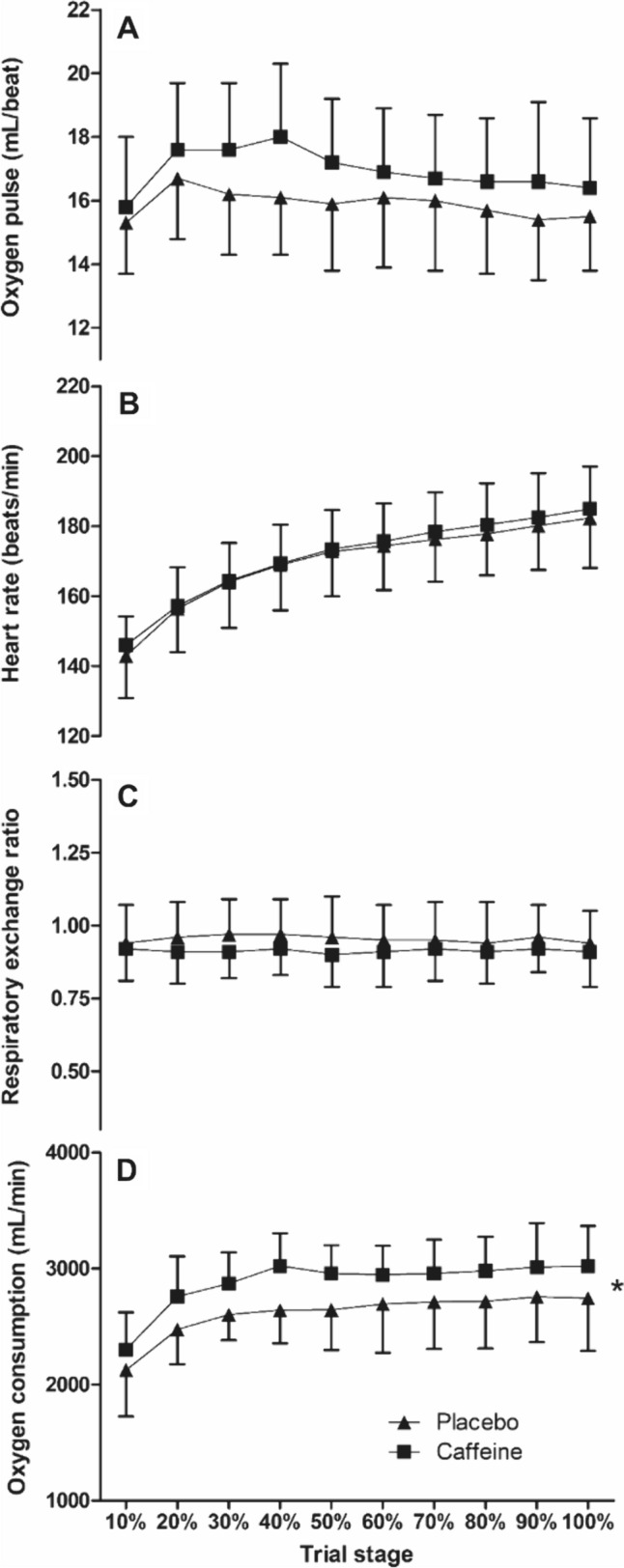
Oxygen consumption (**D**), respiratory-exchange ratio (**C**), heart rate (**B**) and oxygen pulse (**A**) during the time-to-exhaustion trial at the gas exchange threshold during exercise in the heat between caffeine and placebo conditions (mean ± SD). * = condition effect, denoting higher in caffeine (*P* < 0.05)

There was a main effect of the supplement on $\dot{V}$ O_2_ across the TTE (*F*_(1,11)_ = 13.7, *P* = 0.003); however, there was no interaction effect (*P* > 0.05). The mean difference between conditions (caffeine = 2883 ± 219 mL/min *vs.* placebo = 2678 ± 190 mL/min) equated to a 7.4% greater $\dot{V}$ O_2_ across the TTE in the caffeine condition (*d* = 0.78; Fig. 2). The fraction of the thermoneutral $\dot{V}$ O_2peak_ sustained across the placebo trials was 70 ± 3%, whilst the caffeine trial was 74 ± 3%. There were no condition or interaction effects for RER or heart rate (*P* > 0.05; Fig. 2). The O_2_ pulse was not different between conditions (*F*_(1,11)_ = 3.7, *P* = 0.082), despite the 6.2% higher (*d* = 0.49) values following caffeine supplementation compared to placebo (16.9 ± 2.14 mL/beat *vs.* 15.9 ± 1.9 mL/beat; Fig. 2). There were no condition × time interaction effects found across all measures (*P* > 0.05).

There were no main effects of the supplement on glucose oxidation (*F*_(1,11)_ = 0.5, *P* = 0.481) or fat oxidation (*F*_(1,11)_ = 1.5, *P* = 0.240). There was also no interaction with time for glucose oxidation (*P* > 0.05) or fat oxidation (*P* > 0.05). The mean glucose oxidation for caffeine *vs.* placebo (2.5 ± 1.2 g/min *vs.* 2.7 ± 1.3 g/min, respectively; *d* = 0.2) and fat oxidation (0.5 ± 0.4 g/min *vs.* 0.4 ± 0.3 g/min, respectively; *d* = 0.2).

### Thermoregulatory responses

**Fig. 3:**
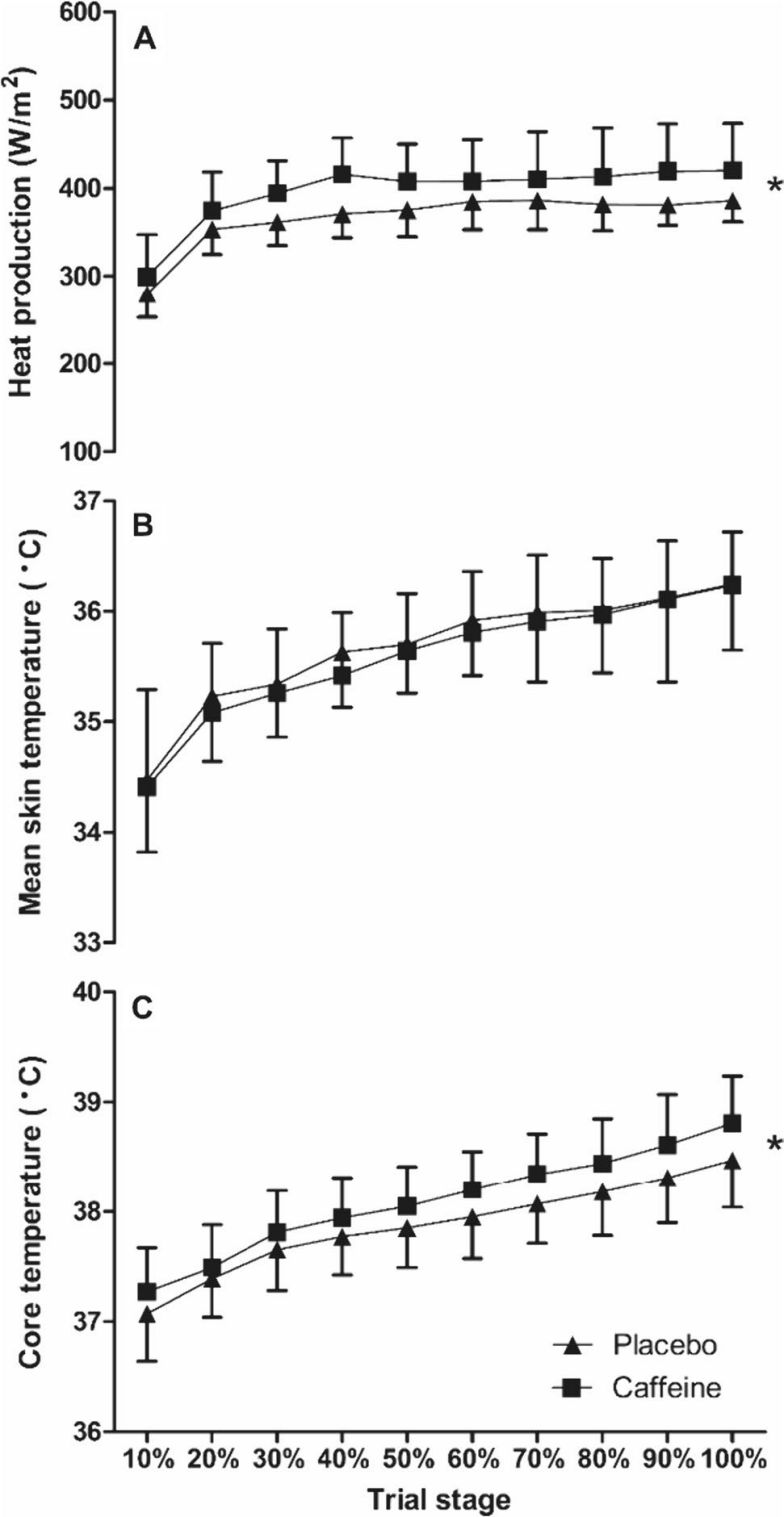
Core temperature (**C**), mean skin temperature (**B**) and heat production (**A**) during the time-to-exhaustion trial in the heat between caffeine and placebo conditions (mean ± SD). * = condition effect, denoting higher in caffeine (*P* < 0.05)

**Fig. 4:**
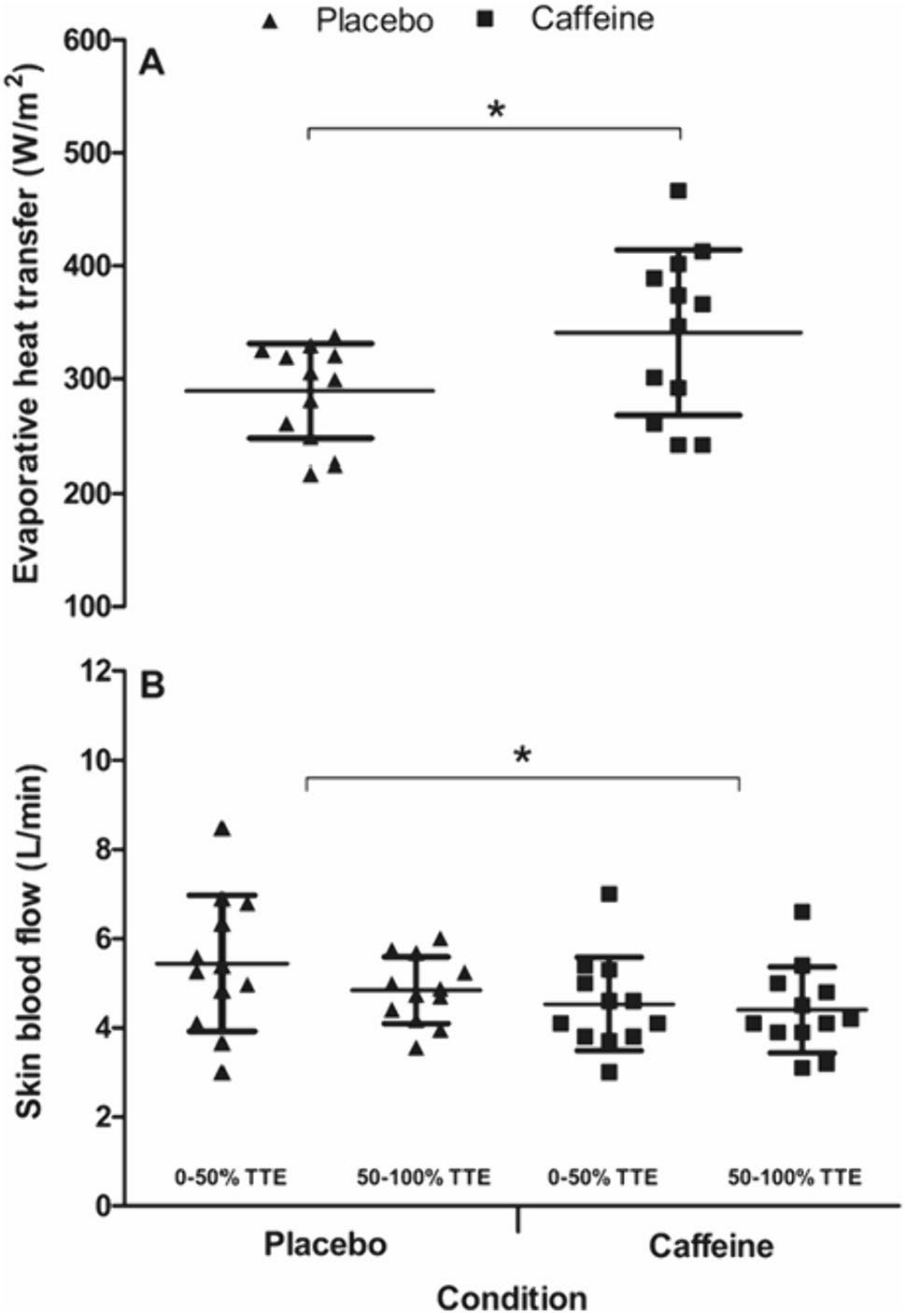
Evaporative heat transfer (**A**) and skin blood flow (**B**) during the time-to-exhaustion trial in the heat between caffeine and placebo conditions (mean ± SD). * = condition effect (*P* < 0.05)

Despite the fixed mechanical intensity, there were main effects of the supplement on $\dot{H}$_prod_ (*F*_(1,11)_ = 12.9, *P* = 0.004), which equated to a 7.9% increase in $\dot{H}$_prod_ in the caffeine *vs.* the placebo condition (*d* = 0.74; Fig. 3). As presented in Fig. 3, there was a main effect of condition on *T*_core_ (*F*_(1,11)_ = 8.8, *P* = 0.013), but no interaction with time (*P* > 0.05). The caffeine condition, therefore, had a significantly (0.6%) higher mean *T*_core_ across the TTE trials compared to placebo (38.09 ± 0.39 °C *vs.* 37.87 ± 0.38 °C, respectively; *d* = 0.58). There was no effect of the condition on *T*_skin_ (*F*_(1,11)_ = 0.2, *P* = 0.701) and no interaction effects with time (*P* > 0.05; Fig. 3). Baseline *T*_core_ (*t*_(11)_ = − 1.5, *P* = 0.159) the rate of rise in *T*_core_ (*t*_(11)_ = − 2.2, *P* = 0.053) was not different between conditions (baseline *T*_core_ placebo = 36.95 ± 0.44 °C *vs.* caffeine 37.07 ± 0.37 °C; *d* = 0.28 and rate of rise in *T*_core_ placebo = 0.02 ± 0.08 °C/h *vs.* caffeine 0.09 ± 0.06 °C/h; *d* = 1.0). WBSR across the whole trial was 21% higher (*t*_(11)_ = 3.2, *P* = 0.008) in the caffeine *vs.* the placebo condition (17.1 ± 4.1 *vs.* 13.8 ± 2.7 mL/min, respectively; *d* = 0.94). The greater sweating response translated to a 16.5% increase $\dot{E}$_sk_ (*t*_(11)_ = 3.0, *P* = 0.006) in the caffeine *vs.* the placebo condition (341 ± 73 *vs.* 289 ± 42 W/m^2^, respectively; *d* = 0.87; Fig. 4) and there were condition effects found for estimated SkBF (*F*_(1,11)_ = 22.2, *P* < 0.001), with the mean SkBF in the caffeine condition 14.1% lower than placebo (4.47 ± 1.01 *vs.* 5.1 ± 1.14 L/min, respectively; *d* = 0.63; Fig. 4).

### Perceptual responses

There was a main effect of condition on TC (*F*_(1,2)_ = 6.2, *P* = 0.029) but no interaction effects (*P* > 0.05). The mean TC was 18.3% higher across the TTE in the caffeine condition compared to placebo (3 ± 1 *vs.* 2 ± 1, respectively; *d* = 0.72). There were no condition or interaction effects for RPE (*P* > 0.05).

## Discussion

The current study aimed to examine the effects of acute caffeine supplementation (5 mg/kg) on thermoregulatory responses and performance during a TTE trial in the heat. Compared to placebo, ingesting caffeine 60 min before exercise resulted in a greater $\dot{V}$ O_2_ response throughout the trial. This notable increase in $\dot{V}$ O_2_, when exercise was controlled at the thermoneutral GET, was sufficient to drive endogenous metabolic heat production (i.e. $\dot{H}$_prod_) 7.9% (*d* = 0.74) higher in the caffeine condition. This is a notable outcome, as based on these results, greater heat storage is therefore inevitable following caffeine ingestion, unless dissipative pathways can compensate for this metabolic heat gain. However, given the increases (0.6%; *d* = 0.58) in *T*_core_, coupled with the attenuated SkBF (− 14.1%; *d* = 0.63) in the caffeine *vs.* placebo condition, it is apparent that thermoregulation was compromised when caffeine was ingested prior to exercising in the heat and thermal comfort was decreased. Interestingly, despite the greater thermal gain in the caffeine condition, there was no difference in TTE, thereby rejecting our primary hypothesis.

We are the first to observe a sustained increase in pulmonary $\dot{V}$ O_2_ when exercising in the heat at pre-determined thermoneutral GET following caffeine ingestion. Interestingly, the increase in $\dot{V}$ O_2_ during the TTE was noted in the absence of any reduction in RER, which is in contrast to a recent meta-analysis of studies with fixed-intensity control (Collado-Mateo et al. 2020) and might be attributed to the addition of an environmental stressor. However, the ~ 7% higher $\dot{V}$ O_2_ recorded during the caffeine trial is comparable to a previous study (Damirchi et al. 2009), where no change in RER was also reported. Indeed, across a number of studies measuring endurance performance after caffeine supplementation in the heat, numerical increases in the $\dot{V}$ O_2_ response to exercise of approximately 3–15% have been reported (Falk et al. 1990; Millard-Stafford et al. 2007; Ping et al. 2010; Pitchford et al. 2014), yet others have demonstrated negligible differences (Beaumont and James 2017; Cheuvront et al. 2009; Roti et al. 2006). However, the methodological differences between studies, such as the mode of performance assessment, intensity and the environmental conditions limits the interpretation of these findings. Similarly, increases in $\dot{V}$ O_2_ have been reported across some studies conducted after caffeine supplementation in thermoneutral conditions (Bell and McLellan 2002; Engels et al. 1999). The null effects of caffeine on RER and substrate oxidation that we report herein indicate an equivalence in substrate metabolism (carbohydrate *vs.* lipids), therefore, consideration of other potential causes of increased $\dot{V}$ O_2_ following caffeine ingestion is necessary_._ For instance, caffeine can raise circulatory catecholamine levels, which has a well-established role in facilitating thermogenesis and the associated ATP demand (Webber and MacDonald 1993), including increases in myocardial oxygen consumption (Robertson et al. 1978; Vasu et al. 1978). Caffeine also exerts an inotropic effect on the myocardium, specifically altering calcium ion release from sarcoplasmic reticulum, which might also lead to greater ATP demand (Rousseau and Meissner 1989). This is consistent with the reported enhancement of left ventricular function following consumption of caffeine-containing beverages (Menci et al. 2013), which others have shown translated to higher stroke volumes (Baum and Weiss 2001). The increase in stroke volume is also consistent with our current finding of reduced peripheral blood flow, and assumed central blood flow redistribution, alongside the indication of higher O_2_ pulse in the caffeine condition (a surrogate marker of stroke volume; Bhambhani et al. 1994). Collectively, there is sufficient reasoning to support the reported increase in the energy cost of exercise in the heat following 5 mg/kg caffeine consumption.

In the current fixed-workload exercise model, the greater $\dot{H}$_prod_ found in the caffeine condition is a direct consequence of the elevated $\dot{V}$ O_2_. Likewise, a natural consequence of increased $\dot{H}$_prod_ would be an increased demand on heat-loss mechanisms, in an attempt to abate elevations in *T*_core_ (Gagge and Gonzalez 1996). Indeed, $\dot{H}$_prod_ is a well-established driver of thermal sweating (Cramer and Jay 2014, 2016; Gagnon and Kenny 2012; Peel et al. 2022), which provides clear reasoning for the greater WBSR with caffeine supplementation compared to placebo. This relationship between $\dot{H}$_prod_ and $\dot{V}$ O_2_ provides a basis for matched $\dot{H}$_prod_ exercise protocols in between-group studies, which was effectively utilised to study the impact of caffeine on thermoregulation in the heat (Hunt et al. 2021). Whilst this model of exercise does not permit direct assessment of performance or capacity, as a result of matching $\dot{H}$_prod_ during exercise, Hunt et al. (2021) reported no effect of caffeine on sweating responses. Therefore, we primarily attribute the increased-sweating response in the caffeine condition to the increased $\dot{H}$_prod,_ but do not rule out the possibility that caffeine may have also directly affected sudomotor responses via increased-sympathetic nervous system activation and release of acetylcholine (Kwon et al. 2022), which is a key regulator of sweat production (Hu et al. 2018). Indeed, ingestion of caffeine prior to exercise in thermoneutral conditions has been reported to significantly increase sweat gland activity and output (Kim et al. 2010, 2011). However, it is currently unclear whether changes in sudomotor activity are the result of caffeine’s affect upon the sympathetic cholinergic sudomotor system (Kwon et al. 2022) or a consequence of a thermogenic effect of caffeine.

Cutaneous vasodilation and the ensuing increase in skin blood perfusion are the most important avenue for convective heat transfer, rising up to 7 L/min during exercise in the heat (Rowell 1974). Interestingly, we observed a 0.63 L/min reduction in estimated SkBF during the caffeine trial, which is in agreement with a recent study (Hunt et al. 2021). Caffeine’s vasoconstrictive effects on blood flow have been reported at the level of macro- and micro- vasculature at rest (Papamichael et al. 2005; Tesselaar et al. 2017) and during exercise (Daniels et al. 1998). The altered haemodynamic findings have been primarily attributed to caffeine’s antagonistic effect on adenosine receptors—mainly A_1_ and A_2_ sub-types (Fredholm 1995). In particular, A_2a_ receptors are predominantly distributed in the smooth muscles of the vasculature and play a vital role in modulating vasomotor tone by facilitating endothelial release of nitric oxide (Hein et al. 1999; Khayat and Nayeem 2017). More importantly, this receptor sub-type is considered to be the main target for caffeine antagonism (see Jacobson et al. 2022), and might explain the observed-peripheral vasoconstriction/reduced vasodilation in the current study. In contrast, a previous study by Stebbins et al. (2001), reported no acute vasoconstrictive effects of caffeine when exercising in the heat, which was explained by a greater net vasodilatory signal via heat-mediated efferent neurons. Although, it was highlighted that participants exercised at varying intensities (50–55% of $\dot{V}$ O_2max_; Stebbins et al. 2001), which would have altered $\dot{H}$_prod_ and, thus, produced uncertainty in the measured-outcome variables, both between conditions (caffeine *vs* placebo) and within-participants.

The greater $\dot{H}$_prod_, accompanied by a subsequent reduction in SkBF, resulted in greater heat being retained rather than lost in the caffeine condition. Therefore, *T*_core_ was ~ 0.2 °C higher in the caffeine trial compared to placebo. In addition to this, there was a non-significant greater rate of rise in *T*_core_ prior to the TTE but after caffeine ingestion, which indicates that the ingestion of caffeine alone, during rest, was sufficient to initiate a *T*_core_ response, perhaps via some of the aforementioned mechanisms. Collectively, these findings are in agreement with two recent meta-analyses (Naulleau et al. 2022; Peel et al. 2021), confirming the negative effect of caffeine on overall heat balance. In the current study, the rise in *T*_core_ was observed despite a 16.5% greater $\dot{E}$_sk_ in the caffeine condition compared to placebo, indicating a mismatch in heat gain *vs.* loss mechanisms. Theoretically, a higher $\dot{H}$_prod_ and *T*_core_ would evoke a greater thermoeffector response, which is supported by the findings of the current study i.e. greater WBSR and $\dot{E}$ sk. The reductions in SkBF that occurred in the caffeine condition alongside increased sweat rates are consistent with the reported disproportionality between these effector responses during exercise in the heat (Cramer et al. 2017). In addition, the vasoconstrictive effects of caffeine and subsequent impact upon dry heat losses, might have further increased the requirement upon eccrine sweat glands recruitment to support sweat production and $\dot{E}$_sk_ (Baker 2019; Green et al. 2004). It is possible that the sustained vasoconstrictive action of caffeine on the skin microvasculature and elevated-sudomotor activity were partly responsible for generating greater systemic metabolic demand, making these a potential source of higher $\dot{V}$ O_2_ requirement, despite the similar external work demand between conditions. It is possible that the lack of convective cooling, owing to the limited air flow in the controlled laboratory, reduced the ecological validity and capacity of both convective and evaporative cooling amongst both conditions. Higher rates of convective (fan) cooling would be a useful addition to future research and, given that an apparent peripheral vasoconstriction and increased sweat response was demonstrated in the caffeine condition, it is uncertain whether this would enhance thermoregulation. It would also be useful for future studies to understand how these changes in whole-body metabolism, O_2_ pulse and the subcutaneous vasculature in the heat are affecting other factors, such as local skeletal muscle blood flow and metabolism, since we cannot confirm here exactly how caffeine ingestion is driving $\dot{H}$_prod._ Furthermore, the worsening in thermal comfort scores perceived during the caffeine trial is indicative of a mismatch between $\dot{H}$_prod_ and heat loss (Epstein and Moran 2006), resulting in participants feeling “hotter” despite controlling for environment between conditions and there being no change in exercise capacity. Interestingly, caffeine had no effect on RPE during constant-work rate cycling in the heat, with similar null effects reported when controlling for $\dot{H}$_prod_ (Hunt et al. 2021).

We attribute the lack of exercise capacity change in the TTE to the negative thermoregulatory effects caused by the caffeine supplementation. However, direct comparison to previous studies is difficult due to varying environmental conditions, performance tests (constant-work rate *vs.* time trial) and exercise intensities utilised (Ely et al. 2011; Ganio et al. 2011; Juan et al. 2009; Roelands et al. 2011; Stebbins et al. 2001). It is our contention that the design of exercise trials could be partly responsible for the inconsistent reports on caffeine’s thermo-physiological effects in the heat. With the assumption of constant baseline exercise efficiency, the external workload becomes the primary driver for $\dot{H}$_prod_ and associated thermoregulatory responses (Cramer and Jay 2016). To assess thermoregulatory effects of caffeine, the majority of previous studies employed constant-work rate exercise, based on %$\dot{V}$ O_2max,_ prior to performing time trials in the heat. Accordingly, some reported higher *T*_core_ values (Cheuvront et al. 2009; Roelands et al. 2011), whilst others showed no thermogenic effect of caffeine in the heat (Beaumont and James 2017; Ganio et al. 2011). The contrasting findings may be related to the %$\dot{V}$ O_2max_ method used for selecting exercise intensity, which does not account for the physiological domains of exercise and is an inaccurate method of exercise prescription between individuals (Iannetta et al. 2020). In addition, the upper aerobic ceiling (i.e. $\dot{V}$ O_2max_) can be reduced by 10–20% when tested in the heat (González-Alonso and Calbet 2003; Lafrenz et al. 2008), which could potentially exacerbate the heterogeneity in metabolic stimulus when using %$\dot{V}$ O_2max_. In the current study, we prescribed exercise intensity at the thermoneutral GET, with the aim of creating a more homogenous metabolic demand between participants (Lansley et al. 2011). Here, the GET occurred at ~ 61% of $\dot{V}$ O_2peak_ in the preliminary incremental ramp test performed in thermoneutral conditions, yet this same external workload elicited a $\dot{V}$ O_2_ response equivalent to ~ 70% and ~ 74% $\dot{V}$ O_2peak_ in the placebo and caffeine trials, respectively, owing to the change in ambient temperature. Whilst $\dot{V}$ O_2_ was, therefore, free to vary using the current study’s methods, the initial domain-based determination of exercise intensity ensured that all participants were equal to, or above, the moderate-to-heavy threshold when commencing the TTE, which is not the case for the commonly adopted arbitrary %$\dot{V}$ O_2peak/max_ methods. Despite the increase in relative intensity compared to the pre-determined values, there was a steady-state $\dot{V}$ O_2_ response, which is associated with exercise < severe domain (Burnley and Jones 2007) and indicates that volitional exhaustion (TTE ~ 30 min) occurred earlier than anticipated across conditions. However, the rate of rise in *T*_core_ amongst the current unacclimated and recreational participants, alongside the numerous other physiological changes ($\dot{V}$ O_2_, HR, RPE, *T*_skin_) was seemingly sufficient to encourage early cessation of exercise. These changes reflect a central integration of the metabolic, cardiovascular and thermal stress experienced by participants, which is likely to have led to a down-regulation of cycling intensity and eventual cessation of exercise, which often occurs in anticipation of any severe homeostatic derangement in the heat (Amann 2011; Nybo 2008; Tucker et al. 2004). Furthermore, the participants included were habituated caffeine consumers, which would also render them more susceptible to thermal strain in the current conditions (Hunt et al. 2021). Based on the current data, further investigation of the domain-based method for determining the effects of supplements on TTE in the heat is warranted.

The amount of caffeine supplemented in the current study (5 mg/kg) is an established-ergogenic dose but, based on the current results, and those reported meta-analytically (Peel et al. 2021), this cannot be assumed in thermally stressful conditions. We have previously reported a range of performance and thermoregulatory responses to caffeine in the heat across nine studies, which included caffeine doses between 3 and 6 mg/kg (Peel et al. 2021). However, it is feasible that smaller doses (1–2 mg/kg; Grgic 2022) of caffeine could confer an ergogenic effect in the heat without the thermogenic effects but, to the best of the authors’ knowledge, doses of < 3 mg/kg have not been investigated. Similarly, it is important that research is conducted to understand whether smaller doses (< 3 mg/kg) of caffeine elicit the same thermogenic effects reported in the current study.

## Conclusion

The current study observed no ergogenic benefit of caffeine supplementation prior to completing constant-work rate exercise in the heat. In contrast, a significant thermogenic effect of caffeine was found, which was accompanied by a reduction in SkBF and a concomitant increase in sudomotor output. The altered metabolic and thermoregulatory responses with caffeine resulted in positive-heat storage and, consequently, a greater elevation in *T*_core_. The caffeine-induced thermo-physiological effects were also perceived by participants, where less thermal comfort was reported compared to the placebo condition. Our findings question the use of caffeine when performing exercise in the heat. Furthermore, these results raise safety concerns for recreational athletes competing in the heat, whereby an ergogenic dose of caffeine might increase the risk of exertional heat illness.

## Data Availability

Data are available upon suitable request.

## Abbreviations

$\dot{{\text{E}}}$_sk_: Evaporative heat transfer
$\dot{{\text{H}}}$_prod_: Metabolic heat production
$\dot{{\text{V}}}$ CO_2_: Pulmonary carbon dioxide production
$\dot{{\text{V}}}$ O_2_: Pulmonary oxygen uptake
$\dot{{\text{V}}}$ O_2peak_: Peak oxygen uptake
$$\dot{{\text{W}}}$$ k: Mechanical work
BSA: Body surface area
GET: Gas exchange threshold
HR: Heart rate
Ṁ: Metabolic energy expenditure
O_2_ pulse: Oxygen pulse
RER: Respiratory exchange ratio
RH: Relative humidity
RPE: Rate of perceived exertion
SH: Specific heat of blood
SkBF: Skin blood flow
*T*_arm_: Mid-humerus temperature
*T*_calf_: Mid -calf temperature
*T*_chest_: Upper chest temperature
*T*_core_: Core temperature
*T*_skin_: Skin temperature
*T*_thigh_: Mid-thigh temperature
TC: Thermal comfort
WBSR: Whole-body sweat rate
